# Endothelial cell source dictates the expression and release of fibrinolytic markers in a proinflammatory environment

**DOI:** 10.1016/j.rpth.2025.102929

**Published:** 2025-06-11

**Authors:** Steven J. Humphreys, Nicola J. Mutch, Claire S. Whyte

**Affiliations:** Aberdeen Cardiovascular and Diabetes Centre, Institute of Medical Sciences, School of Medicine, Medical Sciences and Nutrition, University of Aberdeen, Aberdeen, UK

**Keywords:** coronary artery, endothelial cells, fibrinolysis, shear, thromboinflammation

## Abstract

**Background:**

Endothelial cells (ECs) provide a surface for molecular interactions, secreting various factors that govern hemostasis. Inflammatory cytokines can perturb the vascular microenvironment, potentially causing endothelial dysfunction and dysregulation of hemostasis.

**Objectives:**

To examine the fibrinolytic balance of ECs from different vascular beds and their response to proinflammatory stimuli.

**Methods:**

Primary human umbilical vein ECs (HUVECs), human coronary artery ECs (HCAECs), and immortalized EA.hy926 cells were cultured under venous (2.5 dyne/cm^2^) and arterial (12 dyne/cm^2^) shear stress. ECs were stimulated with thrombin, interleukin 6, or tumor necrosis factor (TNF)-α for 24 hours. The expression of coagulation and fibrinolytic proteins was quantified by quantitative polymerase chain reaction and secreted proteins via ELISA or activity assay. Plasma clot lysis on ECs ± 300 pM tissue-type plasminogen activator (tPA) was monitored at 405 nm.

**Results:**

Basal secretion of C-reactive protein, urokinase plasminogen activator (uPA), and plasminogen activator inhibitor-1 (PAI-1) was higher in HCAECs than in HUVECs, but tPA was similar in both. TNF-α stimulation of HCAECs increased secretion of tPA, uPA, and PAI-1. Levels of tPA/PAI-1 and uPA/PAI-1 were higher in media, as was free-active PAI-1. In contrast, stimulation of HUVECs did not significantly alter gene/protein levels. HCAECs and HUVECs delayed clot lysis relative to no-cell controls by 11 ± 8 minutes (*P* < .01) and 8 ± 6 minutes (*P* < .05) respectively, but were normalized by neutralizing PAI-1. TNF-α stimulation of HCAECs prolonged clot lysis in a PAI-1–dependent manner.

**Conclusion:**

HCAECs respond more potently to a proinflammatory environment than HUVECs, altering expression of fibrinolytic proteins and promoting a hypofibrinolytic response. These data highlight HCAECs as a model of coronary vasculature with potential for screening novel antithrombotic strategies.

## Essentials

• ECs supply key regulators of coagulation and fibrinolysis.
• We compared ECs of venous and arterial origin for their response to flow rate and inflammation.
• Arterial and venous ECs elongated and remodeled differentially in response to flow rate.
• Arterial ECs were more responsive to inflammation, significantly delaying clot breakdown.

## Graphical abstract

**Figure:**
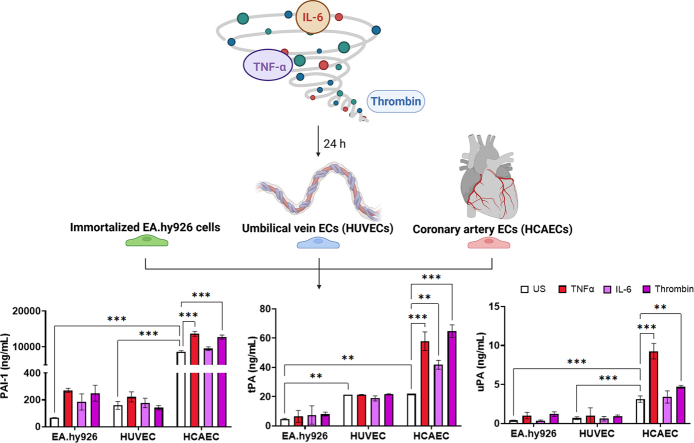

## 1 Introduction

The balance of thrombus formation and dissolution is crucial for maintaining blood fluidity in the vasculature. During normal hemostasis, the equilibrium between the coagulation and fibrinolytic systems is upheld through a sophisticated interplay between plasma, immune cells, platelets, and endothelial cells (ECs) [1]. The vascular endothelium, a heterogeneous monolayer that lines all blood vessels and organs, assumes a crucial role in overseeing a myriad of functions essential for the regulation of blood flow, barrier integrity, as well as inflammatory and immune responses [2]. ECs of arteries and veins have distinct environments dictated by blood hemodynamics and the vascular bed, thereby influencing the composition of thrombi [3]. Inflammation is known to impact EC function, promoting the expression of adhesion molecules, inflammatory markers, and prothrombotic proteins, thereby creating a thromboinflammatory environment [4].

The endothelial hybridoma cell line, EA.hy926 cells, is widely used as an easily accessible, affordable, and convenient alternative to primary ECs in molecular research but differs in its functional response to physiological stimuli [5,6]. Human umbilical vein ECs (HUVECs) are readily available and easily isolated from pooled donor umbilical cords, making them an accessible and cost-effective choice for research. HUVECs are frequently used as a model system for the study and regulation of EC function and, as such, are well-characterized [7,8]. However, HUVECs are derived from a vascular bed of immune-naïve fetal tissue not found in adults and a low-shear stress environment. As such, they are less reflective of diseased vessels and vascular processes [9]. In contrast, human coronary artery ECs (HCAECs) have been shown to exhibit increased expression of inflammatory and adhesion molecules and a higher overall metabolic rate than HUVECs [[10], [11], [12]]. These characteristics make HCAECs a more relevant model for studying vascular disease. However, HCAECs are considerably more expensive than HUVECs, are derived from a single donor, and are provided at their second passage. This limitation affects the number of potential passages before cellular senescence and the onset of endothelial-to-mesenchymal transition, which must be considered when using them in research.

Inflammation involves a complex interplay between plasma, immune cells, and platelets and contributes to the clearance of bacterial and viral infections. If unregulated, these interactions lead to a state of heightened inflammation and are a driving force in the development of arterial and venous thrombosis, termed thromboinflammation [13]. Thromboinflammation is characterized by endothelial dysfunction, complement system activation, cytokine storm, a hypercoagulable state, and impaired fibrinolysis [[14], [15], [16]]. Cytokine receptors, including the tumor necrosis factor receptors (TNFR1 and TNFR2) and interleukin (IL)-6 receptors, play a critical role in mediating and amplifying these inflammatory responses [17]. This multifaceted response is exacerbated in inflammatory conditions, including COVID-19 and sepsis [18,19]. Acute inflammatory markers such as C-reactive protein (CRP) and proinflammatory cytokines, tumor necrosis factor (TNF)-α and IL-6, are predictive of thromboembolic conditions and disrupt fibrinolysis [[20], [21], [22], [23], [24], [25]]. Endothelial secretion of several fibrinolytic factors is dysregulated in response to inflammation in cardiovascular disease, including plasminogen activator inhibitor-1 (PAI-1), urokinase plasminogen activator (uPA), and tissue-type plasminogen activator (tPA) [26,27].

Herein, we investigated the pivotal role played by the endothelium of different vascular beds in their response to physiological shear stress and inflammatory cytokines using immortalized EA.hy926 cells and the primary cells, HUVECs and HCAECs, and examine their influence in regulating the fibrinolytic response. We determined that EA.hy926 cells fail to respond to physiological flow, proving them to be a poor EC model. Importantly, we show that inflammatory cytokine stimulation, observed in pathogenic infections and inflammatory conditions, results in differential upregulation of tPA, uPA, and PAI-1, depending on the EC source, with distinct differences observed between arterial and venous endothelial cells, demonstrating their importance as fibrinolytic regulators. This study highlights the distinct role of the EC source in the regulation of fibrinolysis and, therefore, thrombosis, and may be beneficial for future therapeutic screening.

## 2 Methods

### 2.1 Blood collection

All blood samples were obtained after approval by the University of Aberdeen College Ethical Review Board (CERB/2017/9/1411) in accordance with the Declaration of Helsinki after obtaining informed written consent. Whole blood was collected from 20 consented healthy subjects in 0.1 mL volume of 0.13 M trisodium citrate (Greiner Bio-One) to create a source of pooled normal plasma [28]. Plasma was collected following centrifugation at 1860 × *g* for 30 minutes at 4 °C and subsequently pooled, aliquoted, and stored at −70 °C until required. Ethical approval did not allow for collection of donor demographics.

### 2.2 EC culture

EA.hy926 cells (American Type Culture Collection), a hybridoma cell line derived from HUVECs and A549 epithelial cells, were cultured in Dulbecco’s modified Eagle medium (Gibco) supplemented with 10% fetal bovine serum (Gibco) and 1% penicillin–streptomycin (Gibco). Commercially obtained pooled HUVECs and single-donor HCAECs were purchased and cultured in EC growth medium 2 (PromoCell GmbH) and used experimentally between passages 2 and 5 [8,29]. Cell viability was determined using ReadyProbes Cell Viability Imaging Kit, Blue/Green (Thermo Fisher Scientific). Cells were seeded in Ibidi μ-slides VI^0.4^, 12- or 96-well plates, or 75 cm^2^ flasks at 37 °C in a humidified atmosphere at 5% CO_2_. Cells were stimulated in serum-free medium (PromoCell GmbH) with IL-6, TNF-α (10 ng/mL; BioLegend), or thrombin (1 U/mL; Enzyme Research Laboratories) for 24 hours. Concentrations and times were selected to observe maximal response following dose and time, and were consistent with previous studies [[29], [30], [31]]. Cells were cultured statically or under flow at venous (2.5 dyne/cm^2^) or arterial (12 dyne/cm^2^) shear stress for 30 hours.

### 2.3 Immunocytochemistry

Ibidi slides were coated with 75 μg/mL human fibronectin (Thermo Fisher Scientific), and cells were seeded at 1 × 10^6^ cells/mL and maintained in basal medium at 37 °C with 5% CO_2_ for 30 hours under static or shear conditions. Media was removed, and cells were fixed in 4% paraformaldehyde for 15 minutes at room temperature (RT). Fixed cells were permeabilized with 0.1% (v/v) Triton X-100 in Dulbecco’s phosphate-buffered saline for 10 minutes at RT and subsequently blocked in 1× animal-free blocking buffer (Cell Signaling) for 30 minutes at RT. Cells were subsequently stained with antibodies for endothelial markers rabbit monoclonal VE-cadherin immunoglobulin (Ig) G (1:100; MA5-29141; Thermo Fisher Scientific), rabbit monoclonal ephrinB2 IgG (1:100; MA5-32740; Thermo Fisher Scientific), rabbit monoclonal EphB4 IgG (1:100; MA5-29247; Thermo Fisher Scientific), and Alexa Fluor 488 goat antirabbit IgG (1:600; ab150077; Abcam). Nuclei were stained by NucRed647 Live (1:1000; R37106; Thermo Fisher Scientific) and F-actin by phalloidin–iFluor 555 Reagent (1:1000; ab176756; Abcam). Images were captured using an EVOS M5000 fluorescent microscope (Thermo Fisher Scientific) with an oil-immersion 60× objective lens.

### 2.4 Plasma clot lysis assay

Plasma clot lysis was performed as previously described [25]. Briefly, clots were formed in the presence or absence of ECs seeded at 5 × 10^4^ cells/mL and incubated overnight at 37 °C and 5% CO_2_. Clots were formed from 30% pooled normal plasma in the presence of 16 μM phospholipids (Rossix) ± 300 pM tPA (alteplase [Actilyse]), ±300 pM tPA (tenecteplase [Metalyse]), and ±1 μg/mL neutralizing monoclonal PAI-1 antibody (8H9D4; a kind gift from Paul Declerck) in Tris-buffered saline with Tween 20 (10 mM Tris, 140 mM NaCl, and 0.01% Tween 20, pH 7.4). Clotting was initiated with 10.6 mM CaCl_2_ and 0.1 U/mL thrombin. Absorbance readings at 405 nm were taken every minute for 4 hours at 37 °C on a Powerwave HT microplate reader (BioTek Instruments).

### 2.5 RNA isolation and quantitative polymerase chain reaction

ECs were seeded at 1.5 × 10^5^ cells/mL into 12-well plates, stimulated, and incubated as above before harvesting cells for RNA. Total RNA was extracted using the RNeasy Plus Mini Kit (QIAGEN) and reverse transcribed using the Reverse Transcriptase Core Kit (Eurogentec). Oligos specific for housekeeping and target genes were obtained from Merck Ltd and are listed in Table. Real-time quantitative polymerase chain reaction was performed on a LightCycler 480 system (Roche Diagnostics) in duplicate using a program of preincubation at 95 °C for 5 minutes, followed by amplification for 45 to 65 cycles of 95 °C for 10 seconds, 60 °C for 10 seconds, and a single 72 °C step for 10 seconds. Fold gene expression change was calculated using the 2^–ΔΔCt^ method [32]. Heatmaps were generated to depict fold change in the expression level of the 12 target genes.

**Table:** List of oligos and sequences.

Gene	Forward oligo 5′ to 3′	Reverse oligo 5′ to 3′
GAPDH	AGCCACATCGCTCAGACAC	CGCCCAATACGACCAAAT
PAI-1	AAGACTCCCTTCCCCGACTC	GGGCGTGGTGAACTCAGTATAG
tPA	TGGTGCTACGTCTTTAAGGCGG	GCTGACCCATTCCCAAAGTAGC
uPA	GGCTTAACTCCAACACGCAAGG	CCTCCTTGGAACGGATCTTCAG
Vitronectin	TGGCTGTCCTTGTTCTCCAGTG	GTGTGCGAAGATTGACTCGGTAG
Thrombomodulin	AGCAAGCCCCACTTATTCCC	GGGTGACTCAGGTGAGTTGG
FgnA	CCGGAAGTGAAGCCGATCATGA	CCTGAAGGATGTGTTTGGAGGAC
VWF	GCAGTGGAGAACAGTGGTG	GTGGCAGCGGGCAAAC
CRP	TCGTGGAGTTCTGGGTAGATGG	TTCCCACCGAAGGAATCCTGCT
IL-6R	GACTGTGCACTTGCTGGTGGAT	ACTTCCTCACCAAGAGCACAGC
PAR-1	TACGCCTCTATCTTGCTCATGAC	TTTGTGGGTCCGAAGCAAAT
TNFR1	TATTGGACTGGTCCCTCACC	GTCATTGTACAAGTAGGTTC
TNFR2	GAACCAGCCACAGGCACCA	ACGATGCAGGTGACATTGAC

CRP, C-reactive protein; IL-6R, interleukin-6 receptor; FgnA, fibrinogen α chain; GAPDH, glyceraldehyde 3-phosphate dehydrogenase; PAI-1, plasminogen activator inhibitor-1; PAR-1; protease-activated receptor 1; TNFR, tumor necrosis factor receptor; tPA, tissue-type plasminogen activator; uPA, urokinase plasminogen activator; VWF, von Willebrand factor.

### 2.6 Protein quantification

ECs were seeded at 1.5 × 10^5^ cells/mL into 12-well plates, stimulated, and incubated as above. Media was collected and centrifuged at 3000 × *g* for 10 minutes at 4 °C prior to storage. Total antigen concentrations of PAI-1, CRP, and uPA in cell media were determined using Simple Plex assays on Ella system, according to manufacturers’ guidelines. Concentrations of tPA, active PAI-1, and plasminogen activator (PA)/PAI-1 complexes were quantified using ELISA kits from Innovative Research, and von Willebrand factor (VWF) from Abcam, according to manufacturers’ guidelines.

### 2.7 Literature review

A thorough review of existing literature was undertaken utilizing the PubMed database with the following search terms: “thrombosis” and “hemostasis,” alongside identifiers specific to ECs, such as “HUVEC,” “HCAEC,” “HAEC,” and “HMVEC.” The scope of the search was restricted to articles published in English over the last decade.

### 2.8 Statistical analysis

Data were analyzed using GraphPad Prism v10. Results are represented as the mean ± SEM or fold-change compared with untreated cells. Experiments were performed in triplicate unless stated otherwise. Significance was determined using a 2-way analysis of variance using Dunnett’s post hoc comparison test with ∗*P* < .05, ∗∗*P* < .01, and ∗∗∗*P* < .001. ImageJ (National Institutes of Health) was used to measure the nucleus aspect ratio. Pixels were converted to microns (60× objective, 40 μm scale = 485 pixels). Nuclei images were transformed to 8-bit, smoothed, converted to binary, separated by watershed, and outlined. Aspect ratio was obtained and exported to Prism.

## 3 Results

### 3.1 Primary ECs maintain their *in vivo* identities

**Figure 1:**
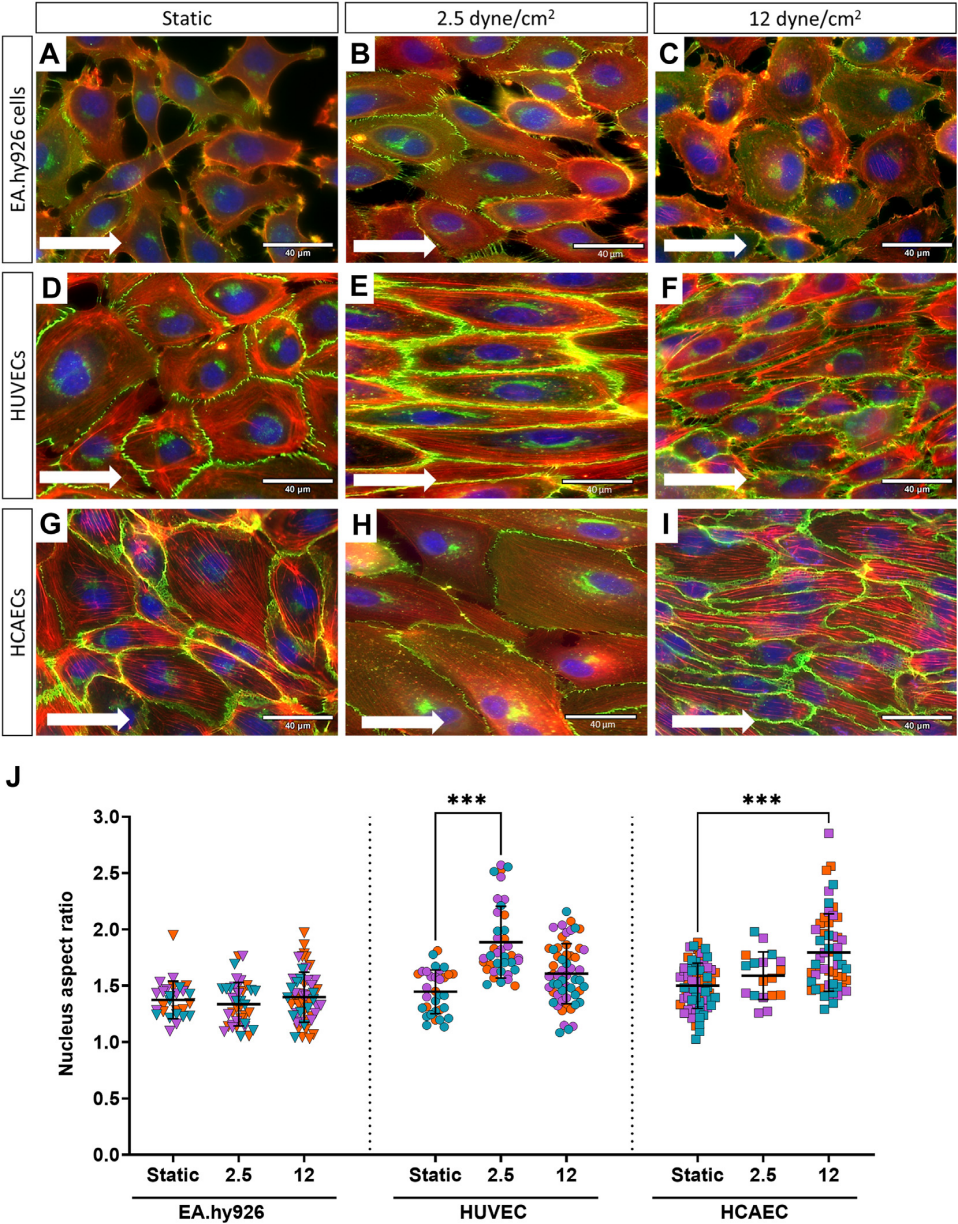
Primary endothelial cells align with flow in a shear-dependent manner. EA.hy926 cells (A–C), human umbilical vein endothelial cells (HUVECs; D–F), and human coronary artery endothelial cells (HCAECs; G–I) were cultured in Ibidi μ-Slide VI Luer 0.4 channel slides for 30 hours at 37 °C with 5% CO_2_ either statically or under flow at venous (2.5 dyne/cm^2^ [B, E, and H]) or arterial (12 dyne/cm^2^ [C, F, and I]) shear stress. Cells were fixed and subsequently stained for VE-cadherin (green), F-actin (red), and nuclei (blue) and imaged on the EVOS M5000 microscope with a 60× oil-immersion objective. Endothelial cell remodeling was measured by the aspect ratio of the nucleus, and the replicates of each experimental repeat were color-coded (J). White arrows indicate the direction of flow. Data represent the mean ± SEM, *n* = 3. ∗*P* < .05, ∗∗*P* < .01, ∗∗∗*P* < .001. Scale bar = 40 μm.

*In vivo,* the endothelium is exposed to constant shear stress. To understand the impact of this on EC response, *in vitro* cells were cultured statically or exposed to similar continuous shear stresses observed in veins (2.5 dyne/cm^2^) or arteries (12 dyne/cm^2^) [33]. Statically cultured EA.hy926 cells (Figure 1A) showed low confluency and a lack of the typical cobblestone morphology found in *in vitro* EC cultures. Extensive lamellipodia and filopodia protrusions from EA.hy926 cells, associated with spreading and migration, were visible; however, there was a distinct lack of actin filaments. When cultured under increasing shear stress (Figure 1B, C), EA.hy926 cells failed to elongate or align with the direction of flow, as evidenced by disorganized actin and discontinuous junctional staining of VE-cadherin. VE-cadherin was also located adjacent to the nuclei under both static and shear conditions, and this was consistent across all 3 cell types (Figure 1A–I). In contrast to EA.hy926 cells, HUVECs exhibited confluency and cobblestone morphology under static culture (Figure 1D). When exposed to venous shear, HUVECs demonstrated clear cell elongation and actin fiber alignment in the direction of flow (Figure 1E). In contrast, arterial shear negatively impacted HUVEC behavior, with cell rounding and disrupted junctional staining observed (Figure 1F). HCAECs cultured under static conditions showed similar morphology and confluency to HUVECs (Figure 1G–I), with minimal shape change upon application of venous shear, although some alignment of actin fibers in the direction of flow was observed. Arterial shear supported the alignment of HCAECs in the direction of flow, with consistent junctional staining between cells and clear cytoskeleton remodeling (Figure 1I). Shear stress induced changes to the nuclear aspect ratio, which is defined as the ratio between the long and short axes of the nucleus. There were no significant differences between the different types of statically cultured ECs (Figure 1J), and shear stress had no effect on EA.hy926 cell nuclei. However, nuclei of HUVECs cultured at venous shear were significantly elongated, and a similar effect was observed in HCAECs cultured under arterial shear stress.

**Figure 2:**
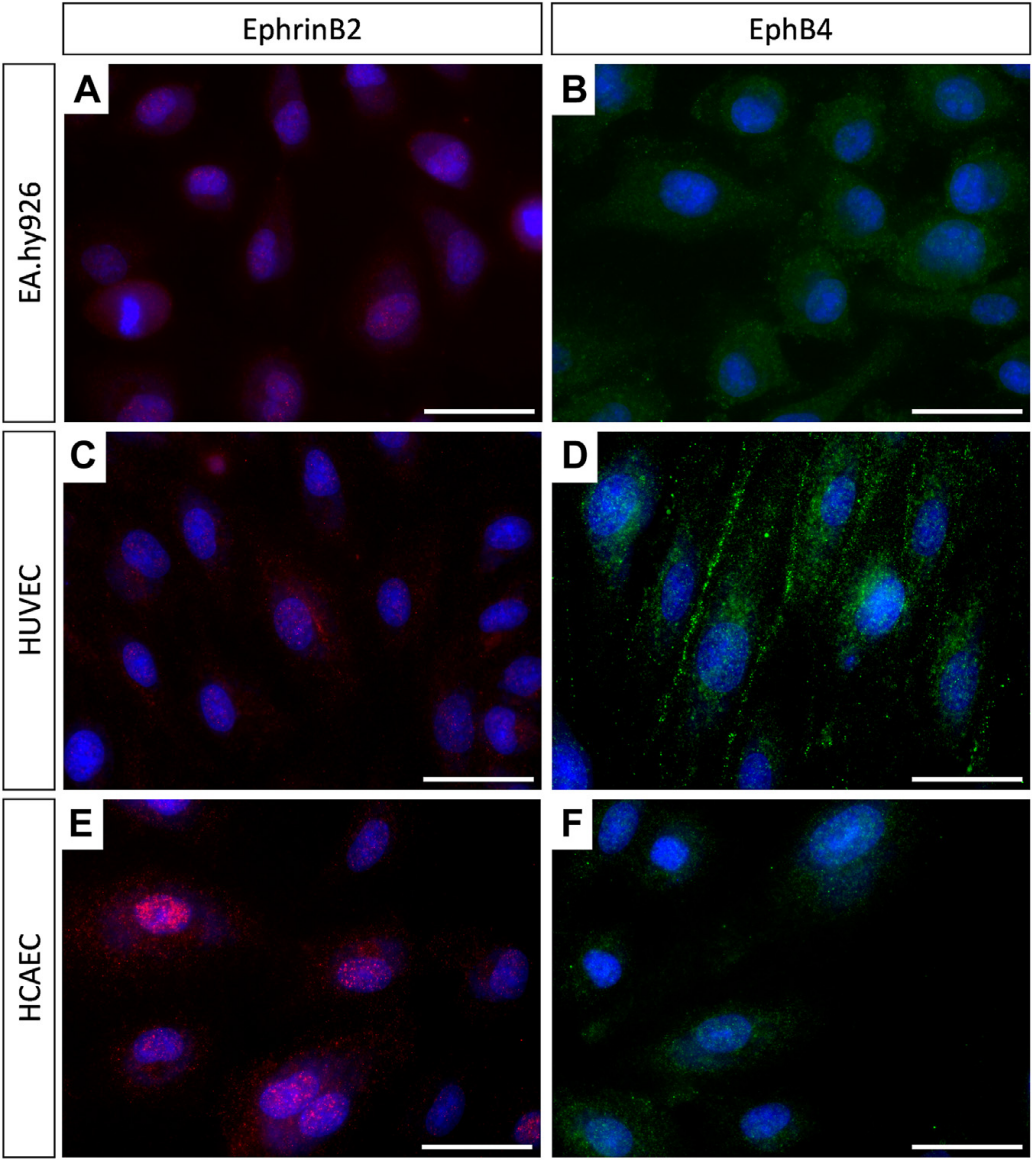
EphrinB2 and EphB4 are differentially expressed in *in vitro* cultured endothelial cells. EA.hy926 cells (A and B), human umbilical vein endothelial cells (HUVECs; C and D), and human coronary artery endothelial cells (HCAECs; E and F) were cultured under static conditions in Ibidi μ-Slide VI Luer 0.4 channel slides for 30 hours at 37 °C with 5% CO_2_. Cells were fixed with paraformaldehyde, permeabilized with Triton X-100, and blocked with a blocking buffer. Cells were incubated with ephrinB2 (red; A, C, and E) or EphB4 (green; B, D, and F) antibodies at 1:100. Detection was visualized using an Alexa Fluor 488-labelled goat antirabbit antibody (1:1000). Nuclei were stained with NucRed647 Live (blue). Cells were imaged on the EVOS M5000 microscope with a 60× oil-immersion objective. Images are representatives of *n* = 3. Scale bar = 40 μm.

To establish whether ECs retain their *in vivo* identities in culture, we examined ephrinB2 and EphB4 as markers of arterial and venous ECs, respectively. EphrinB2 exhibited an elevated level of expression within arterial cells, occurring within both the nucleoplasm and cytoplasm, whereas EphB4 was notably more abundant in venous cells on the cell membrane [34]. Increased cytoplasm and nucleoplasm staining of ephrinB2 (red) was observed in HCAECs compared with EA.hy926 cells and HUVECs (Figure 2A, C, E). Preferential localization of a transmembrane signal of EphB4 (green) was apparent in HUVECs (Figure 2D) with only low-level cytoplasmic staining in EA.hy926 cells and HCAECs (Figure 2B, F). Expression of ephrinB2 and EphB4 was consistent under both static and shear cultured conditions (data not shown).

### 3.2 Endothelial-derived PAI-1 delays plasma clot lysis

**Figure 3:**
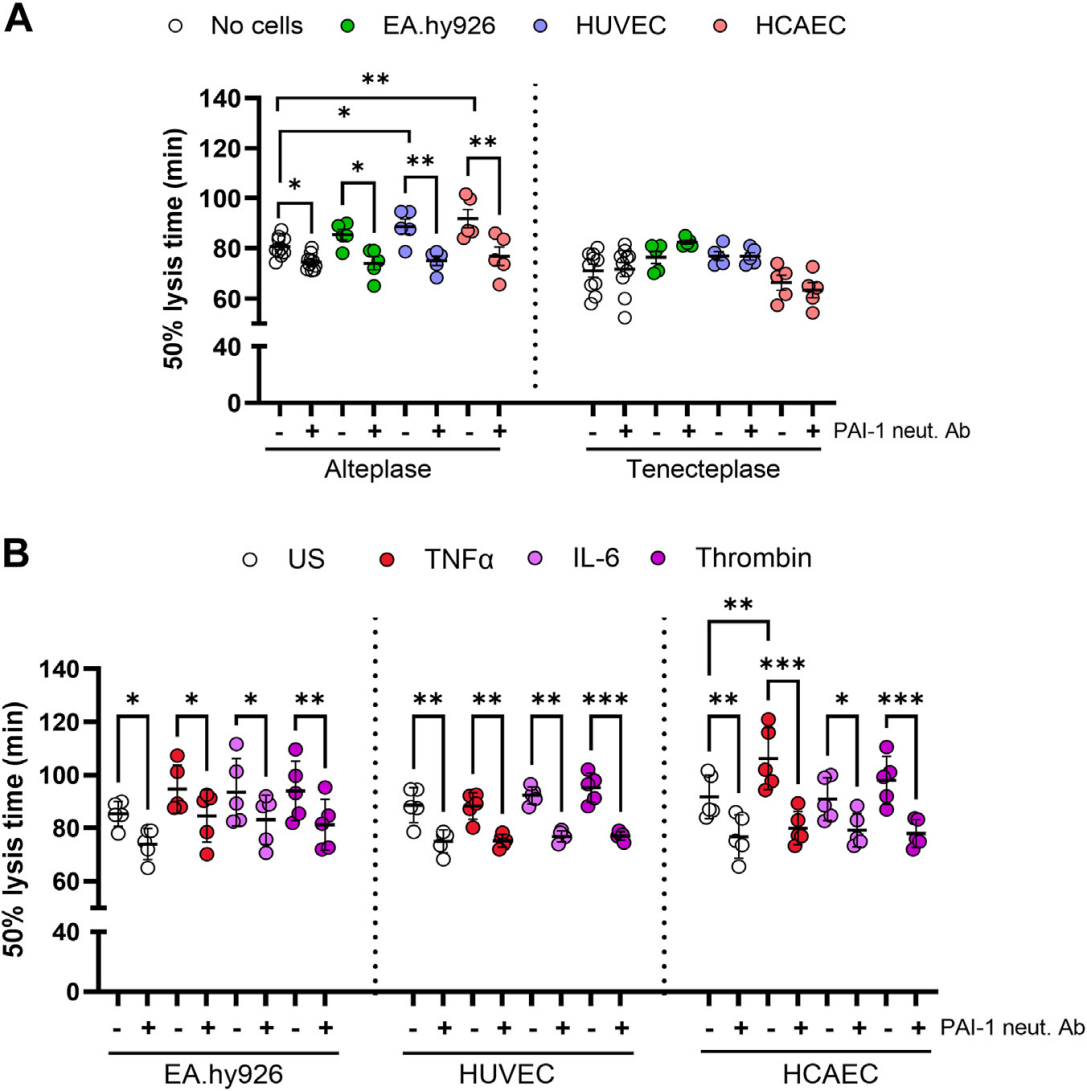
Endothelial-derived plasminogen activator inhibitor-1 (PAI-1) delays plasma clot lysis. Plasma clots were formed in the absence or presence of endothelial cells seeded at 5 × 10^4^ cells/mL and incubated overnight under static conditions at 37 °C and 5% CO_2_ (A). Confluent monolayers of endothelial cells were left unstimulated in serum-free media or stimulated with 10 ng/mL tumor necrosis factor (TNF)-α, interleukin (IL)-6, or 1 IU/mL thrombin for 24 hours (B). Plasma clots consisting of 30% pooled normal plasma, 16 μM phospholipids, ± 300 pM tissue-type plasminogen activator (tenecteplase [A] or alteplase [A and B]), and ± 1 μg/mL of a neutralizing (neut.) PAI-1 antibody (Ab) were clotted with 10.6 mM CaCl_2_ and 0.1 IU/mL thrombin in a microtiter plate in the absence or presence of a confluent monolayer of human umbilical vein endothelial cells (HUVECs) or human coronary artery endothelial cells (HCAECs). Absorbance readings at 405 nm were taken every minute for 4 hours at 37 °C. For comparison, unstimulated endothelial cell data are shown in both panels (A) and (B). Data represent the mean ± SEM, *n* ≥ 5. ∗*P* < .05, ∗∗*P* < .01, ∗∗∗*P* < .001. US, unstimulated.

We addressed the functional impact of ECs in dictating the fibrinolytic response in a tPA-mediated plasma clot lysis assay (Figure 3A). EA.hy926 cells did not significantly alter clot lysis times. In contrast, HUVECs (8 ± 6 minutes; *P* < .05) and HCAECs (11 ± 8 minutes; *P* < .01) significantly delayed clot lysis compared with no-cell control conditions. Inclusion of a neutralizing antibody targeting PAI-1 mitigated the effects of the ECs on clot lysis. A comparable enhancement in fibrinolytic activity was noted when the PAI-1–resistant variant of tPA, tenecteplase, was replaced with alteplase. These data suggest that ECs delay fibrinolysis due to secretion of PAI-1.

To replicate thromboinflammatory complications *in vivo*, ECs were subjected to proinflammatory stimuli (Figure 3B) prior to performing clot lysis (Figure 3A). Stimulation of EA.hy926 cells and HUVECs did not cause a significant change in clot lysis time. In contrast, there was a significant delay in clot lysis following HCAEC stimulation with TNF-α, which was ablated by inclusion of a neutralizing monoclonal antibody to PAI-1. Notably, neither IL-6 nor thrombin stimulation of any cell type further delayed clot lysis compared with the unstimulated ECs.

### 3.3 HCAECs are more sensitive to a proinflammatory environment than HUVECs

**Figure 4:**
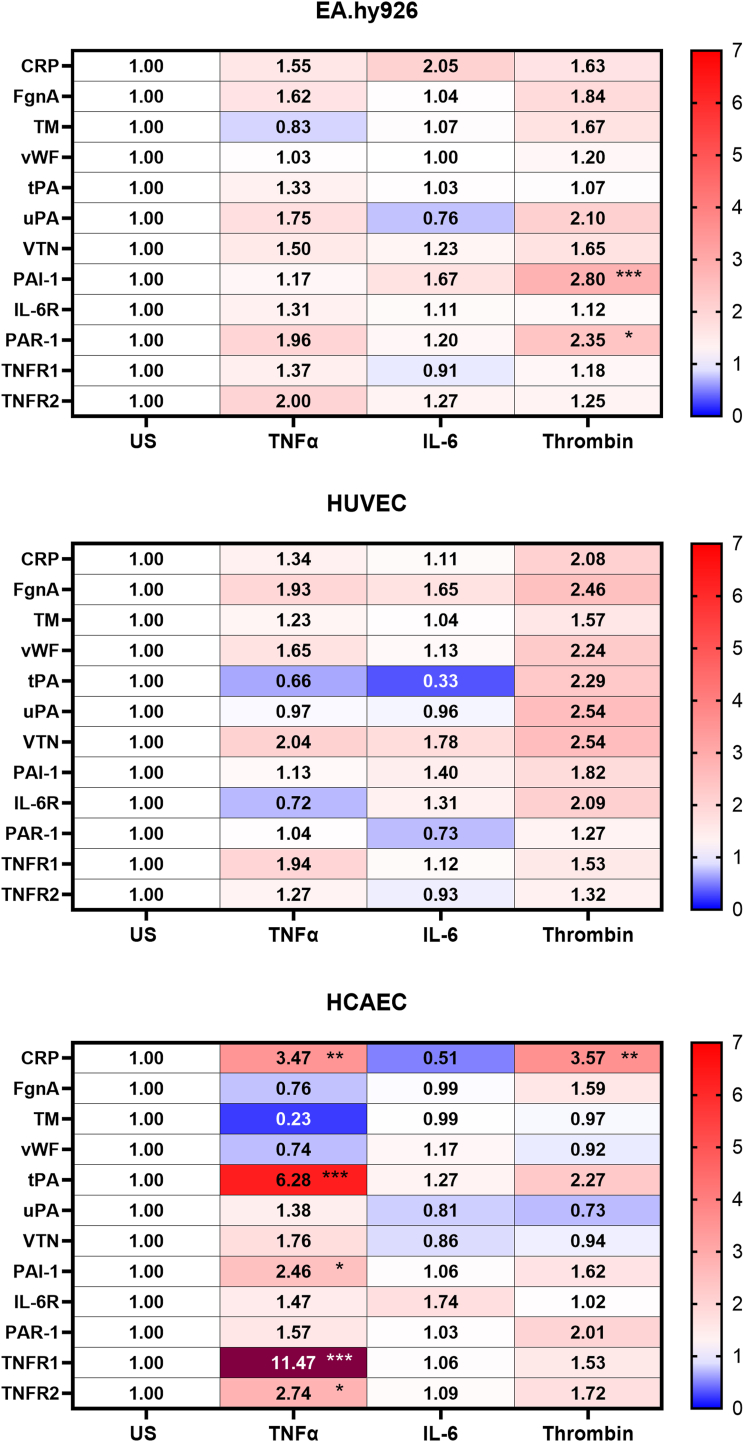
Following endothelial inflammation, inflammatory, endothelial, and fibrinolytic gene expression is altered. Endothelial cells were seeded at 1.5 × 10^5^ cells/mL and incubated overnight under static conditions at 37 °C and 5% CO_2_. The next day, cells were left unstimulated in serum-free media or stimulated with 10 ng/mL tumor necrosis factor (TNF)-α, interleukin (IL)-6, or 1 IU/mL thrombin for 24 hours. Cells were lysed, RNA was extracted, and gene expression was measured by a LightCycler 480 II using Takyon No ROX SYBR 2X MasterMix (Eurogentec). Data represent the mean ± SEM, *n* = 3. ∗*P* < .05, ∗∗*P* < .01, ∗∗∗*P* < .001. CRP, C-reactive protein; FgnA, fibrinogen α chain; HCAEC, human coronary artery endothelial cell; HUVEC, human umbilical vein endothelial cell; PAI-1, plasminogen activator inhibitor-1; PAR, protease-activated receptor; TM, thrombomodulin; TNFR, tumor necrosis factor receptor; tPA, tissue-type plasminogen activator; uPA, urokinase plasminogen activator; US, unstimulated; VTN, vitronectin; VWF, von Willebrand factor.

Upregulation of inflammatory, endothelial, and fibrinolytic genes in HUVECs following cytokine stimulation was minimal (Figure 4). Notably, both TNF-α and IL-6 stimulation downregulated *tPA* gene expression in HUVECs. Interestingly, in contrast to cytokine stimulation, thrombin tended to upregulate the expression of all inflammatory, endothelial, and fibrinolytic markers quantified in HUVECs and EA.hy926 cells. In EA.hy926 cells specifically, thrombin stimulation significantly upregulated *PAI-1* (*P* < .001) and *PAR-1* (*P* < .05). TNF-α stimulation of HCAECs significantly upregulated gene expression of *CRP* (*P* < .01), *tPA* (*P* < .001), *PAI-1* (*P* < .05), *TNFR1* *(P <* .001), and *TNFR2* (*P* < .05), while thrombin upregulated expression of *CRP* only (*P* < .001) compared with unstimulated cells. IL-6 consistently had no significant impact on all genes assessed in all 3 cell types. There were no significant differences in gene expression of *fibrinogen α chain, thrombomodulin, uPA, VWF, vitronectin*, or *IL-6* *receptors*, irrespective of cell type.

**Figure 5:**
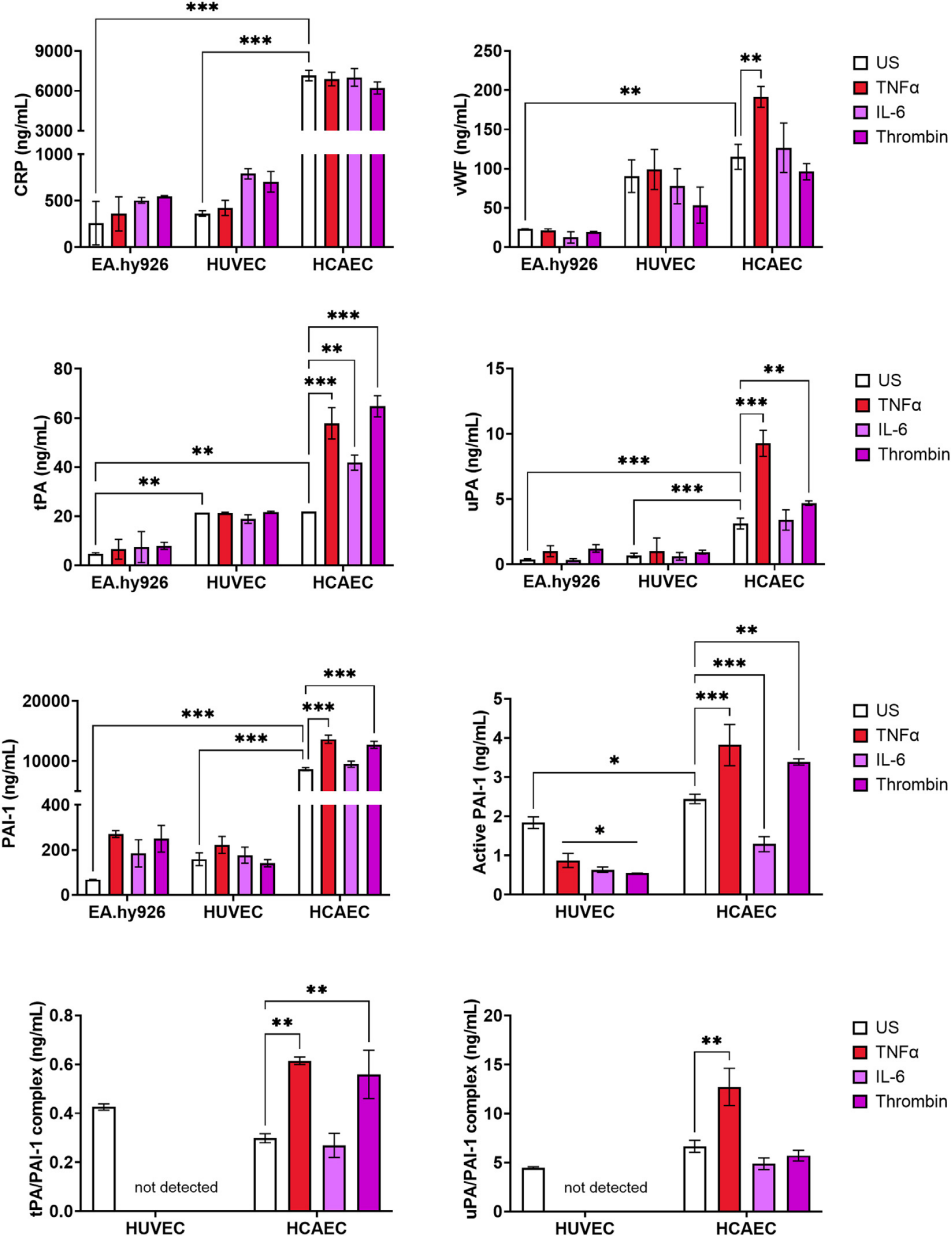
Inflammatory markers and fibrinolytic proteins are elevated in human coronary artery endothelial cells (HCAECs). Endothelial cells were seeded at 1.5 × 10^5^ cells/mL and incubated overnight under static conditions at 37 °C and 5% CO_2_. The next day, cells were left unstimulated or stimulated with 10 ng/mL tumor necrosis factor (TNF-α), interleukin (IL)-6, or 1 IU/mL thrombin for 24 hours. The media were harvested and measured by Simple Plex assays on Ella system or by enzyme-linked immunosorbent assay. Data represent the mean ± SEM, *n* = 3. ∗*P* < .05, ∗∗*P* < .01, ∗∗∗*P* < .001. CRP, C-reactive protein; HUVEC, human umbilical vein endothelial cell; PAI-1, plasminogen activator inhibitor-1; tPA, tissue-type plasminogen activator; uPA, urokinase plasminogen activator; US, unstimulated; VWF, von Willebrand factor.

Strikingly, basal secretion of CRP, PAI-1, and uPA was significantly higher in HCAECs than in EA.hy926 cells and HUVECs (*P* < .001; respectively; Figure 5). Primary cells showed similar basal secretion of VWF and tPA; however, antigen levels in EA.hy926 cells were lower. Stimulation of EA.hy926 cells did not significantly alter protein levels. Similarly, stimulation of HUVECs did not significantly change protein secretion; however, all stimuli led to a significant downregulation of PAI-1 activity (*P* < .05), and PA/PAI-1 complexes were undetectable in HUVECs. TNF-α stimulation of HCAECs significantly upregulated expression and secretion of VWF (*P* < .01), tPA (*P* < .01), uPA (*P* < .001), and PAI-1 (*P* < .001), but not CRP. Significant upregulation of tPA (*P* < .01), uPA (*P* < .01), and PAI-1 (*P* < .001) in HCAECs was also observed with thrombin. Levels of tPA/PAI-1 (*P* < .01) and uPA/PAI-1 (*P* < .001) complexes were higher in the media, as was free-active PAI-1 (*P* < .05) in TNF-α–stimulated HCAECs. These data indicate that TNF-α can induce significant changes in inflammatory, endothelial, and fibrinolytic genes and proteins in HCAECs compared with EA.hy926 cells and HUVECs. These data demonstrate that HCAECs exhibit a heightened responsiveness to thromboinflammatory signals, which may contribute to inflammatory and fibrinolytic processes. These findings underscore the important role of the EC source in modulating inflammation and fibrinolysis within a thromboinflammatory environment.

## 4 Discussion

In this study, we demonstrate the differential response of human ECs from different vascular beds and show HCAECs are more responsive to TNF-α–induced inflammation, which upregulates inflammatory, endothelial, and fibrinolytic markers, thereby promoting a hypofibrinolytic response. ECs are crucial to the regulation of hemostasis, maintaining the equilibrium between a pro- and anticoagulant state. The varying hemodynamic forces, vessel diameters, and structures, ie, branched and unbranched, within the vasculature promote heterogeneity, with distinct differences recorded in endothelial permeability throughout the vasculature [3,4]. Despite this, within the last decade, HUVECs, a naïve cell line used in the study of hemostasis and thrombosis, have become the gold standard, with >500 papers published, compared with 12 using HCAECs, 9 using human aortic ECs, and 13 using human microvascular ECs.

EphrinB2 and EphB4, a ligand and receptor pair, play crucial roles in the differentiation and function of venous and arterial ECs across the vasculature [34]. EphrinB2 is expressed primarily in arterial cells and is involved in signaling between the nucleoplasm and cytoplasm. EphB4 is notably more abundant in venous cells, where it functions as a signal transducer on the cell membrane [35,36]. Primary ECs in culture retain differential markers that are indicative of their origin. In contrast, these are lost in the hybridoma EA.hy926 cell line. However, it has been reported that the differential distribution of ephrinB2 is lost *in vitro* [37]. The shear-dependent alignment and remodeling of HUVEC and HCAEC cell–cell junctions, nuclei, and cytoskeletons, and the differential expression of ephrinB2 and its receptor EphB4 indicate that the primary ECs have retained their *in vivo* identity in culture.

Exposure of ECs to hemodynamic forces shifts their proliferation, migration, morphology, and adherence properties, indicating a rapid and coordinated response [38,39]. We observed the junctional protein VE-cadherin at the cell periphery and adjacent to the nucleus. This phenomenon is consistent with trafficking of VE-cadherin from the Golgi to the cell membrane, which involves the Golgi-associated protein, cPLA2α [40]. We demonstrate that when the primary ECs, HUVECs and HCAECs, are exposed to physiological shear stresses, they elongate and align in the direction of flow in a shear and cell-dependent manner. Physiological shear stress induces rapid actin cytoskeleton remodeling of the endothelium in the direction of flow and alters the expression of junctional proteins. In marked contrast, EA.hy926 cells did not respond to shear stress. Previously, EA.hy926 cells showed different levels of mechanosensor expression of *CDH5, VEGFR2, PIEZO1/2, CCM1/2/3,* and *ITGBP1* compared with HUVECs and human dermal microvascular ECs [6,[41], [42], [43]]. The use of immortalized cell lines offers the advantage of negating the variability associated with primary EC types, making them a valuable tool for high-throughput experiments due to their capacity for unlimited growth and for cost-effective *in vitro* culturing. However, this study shows significant and substantial differences in their response to physiological shear. Additionally, immortalized cells are prone to chromosomal instability, loss of primary EC responses, and lack of the differential endothelial identity retained in the isolated tissue [44].

Our results demonstrate that HUVECs and HCAECs, but not EA.hy926 cells, significantly perturb alteplase-mediated fibrinolysis in a plasma clot lysis assay. Fibrinolytic potential is restored by neutralization of PAI-1, indicating that basal secretion of this inhibitor directly impacts clot stability. When we triggered fibrinolysis with tenecteplase, a variant of alteplase with an 80-fold reduction in sensitivity to PAI-1 [45], it restored the fibrinolytic potential to levels similar to no-cell control. We show that TNF-α stimulation of HCAECs enhanced PAI-1 expression and activity, which caused a further delay in fibrinolysis. Impaired fibrinolysis is associated with a predisposition to coronary artery disease in patients with elevated levels of PAI-1 [11,46,47]. This indicates that HCAECs have a unique responsiveness that could explain why coronary arteries are more susceptible to inflammation and atherogenesis.

TNF-α is a key proinflammatory cytokine involved in a wide array of physiological and pathological processes, including vascular permeability and endothelial activation. Its effects are mediated through 2 distinct membrane-bound receptors: TNFR1 and TNFR2. TNF-α primarily signals through the ubiquitously expressed membrane receptor TNFR1; however, ECs also express TNFR2 [48]. We show that TNF-α stimulation leads to the significant upregulation of both *TNFR1* and *TNFR2* in HCAECs, highlighting an enhanced sensitivity of these cells to proinflammatory signaling. This upregulation of *TNFR1* is consistent with role of this receptor in augmenting early- and late-stage atherosclerosis [49]. While TNFR1 signaling is well-characterized and primarily associated with apoptosis, TNFR2 remains less extensively researched but is reported to play a role in mediating the immune response [50,51]. Interestingly, in this study, our findings show that TNF-α and thrombin stimulation upregulate *CRP* mRNA expression in all 3 EC types; however, this is not reflected in protein secretion. These data may suggest differences in translational regulation relating to variances in intracellular protein degradation and/or secretion or clearance mechanisms. Notably, Chen et al. [52] recorded peak CRP activity at 12 hours in HCAECs, indicating a time-dependent effect, attributing loss of activity to receptor-mediated clearance. Our results indicate that stimulation with TNF-α or thrombin augments fibrinolytic factors, including PAI-1, tPA, and uPA gene expression/protein secretion in HCAECs, a phenomenon linked to the cytokine storm in various pathological states, including stroke, trauma, and COVID-19 [25,[53], [54], [55], [56]]. Pandey et al. [57] demonstrated that while both TNFRs are required for maximal PAI-1 expression during chronic inflammation, TNFR2 acts as a negative regulator in acute TNF-α–induced responses. This may indicate that TNFRs differentially modulate the endothelial fibrinolytic response during TNF-α–induced thromboinflammatory conditions, but further work is necessary to define their exact function and how this response is modulated by an inflammatory challenge. Our observed upregulation of tPA and uPA suggests a compensatory attempt to enhance fibrinolysis in response to elevated levels of PAI-1, which likely acts as a protective mechanism to mitigate the increased risk of thrombosis induced by inflammation [58,59]. This compensatory phenomenon has been clearly demonstrated in inflammatory conditions, including COVID-19, where elevated PAI-1 and impaired fibrinolysis contribute to thrombotic risk [25,60]. While the simultaneous upregulation of total and active PAI-1, observed in this study, indicates a counter-regulatory mechanism, it is this balance between pro- and antifibrinolytic factors that is crucial for maintaining vascular homeostasis, highlighting the crucial role of the vascular bed in maintaining equilibrium.

We found that TNF-α stimulation upregulates the expression of *vitronectin* in all 3 EC types, potentially extending the half-life of active PAI-1 and potentially further exacerbating inhibition of fibrinolysis. This effect is more pronounced in HCAECs, where the increased presence of active PAI-1 may contribute to the prothrombotic environment in the coronary artery [11,61]. We observed a strong correlation between tPA/PAI-1 and uPA/PAI-1 inhibitory complex formation and PAI-1 activity in HCAECs, which may indicate a shift toward a more prothrombotic state, potentially increasing the risk of thrombosis in coronary arteries [11]. In contrast, we found that there were no detectable levels of PA/PAI-1 complexes following TNF-α stimulation of HUVECs and downregulation of PAI-1 activity. These distinctions may stem from the heterogeneity and plasticity of ECs derived from different vascular beds, as seen in differential marker expression and varied responses to physiological shear stress [62,63]. HUVECs represent a more naïve EC type that would not be predisposed to thromboinflammatory conditions [64]. Studies comparing alternative ECs describe disadvantages of HUVECs, indicating they harbor a closer relationship to human-induced pluripotent stem cell-derived ECs than to 5 more mature EC types [9,64]. This is reflected in their limited responsiveness to inflammatory stimuli, failing to recapitulate the inflammatory signaling of ECs from a more mature vascular bed. As a result, HUVECs may not adequately model the thromboinflammatory responses observed *in vivo*, particularly in the context of diseases such as atherosclerosis and coronary artery disease [65].

In this study, we illustrate that shear stress has a significant impact on the elongation, alignment, and remodeling processes of primary ECs. HCAECs exhibit a more robust response to a proinflammatory environment compared with HUVECs, as indicated by the heightened expression and secretion of PAI-1, tPA, and uPA. PAI-1, derived from ECs, exacerbates hypofibrinolysis and is further intensified by proinflammatory triggers in cells of coronary origin. Due to methodological constraints, it was not possible to replicate shear conditions when assessing cytokine responsiveness or fibrinolysis. Therefore, future studies employing dynamic flow systems will be necessary to fully elucidate the impact of shear stress on these processes. Further research is essential to unravel the complex signaling molecules and pathways that drive the functional differences between HUVECs and HCAECs, as well as ECs of other origins. Understanding these differences is crucial for determining the impact of vascular bed-specific ECs in modeling diseases, like coronary artery disease, which specifically affect distinct vascular beds. Given the variable response of ECs to inflammatory stimuli, HCAECs may provide a more representative model for studying arterial thrombotic events, ultimately enabling the development of more precise, targeted strategies for combating these conditions.
